# Arsenic in the Hair

**Published:** 1901-04-13

**Authors:** 


### ARSENIC IN THE HAIR.

WHEN a person is taking arsenic he excretes arsenic, be the same more or less, so that it may be possible to ascertain whether or not arsenical poisoning is actually or has recently been going on by a chemical examination of the urine. It not infrequently, however, becomes a question, not so much whether a person is now being poisoned, as whether certain symptoms, peripheral neuritis, for example, are due to an exposure to arsenical poisoning which may have ceased for some time. As one means of solving this problem it has been shown by Mr. W. Kirkby that, from the predilections of arsenic for the skin, it has been possible to detect it in a scab from a patient's body, and thus to confirm a diagnosis of arsenical neuritis. All patients, however, do not have scabs or scales which can be analysed, but most patients have hair, and it has been shown by Dr. Knecht and Dr. Dearden,[1] of Manchester, that it is possible by chemical examination of one gramme of hair, removed from a patient, to obtain the evidence required. In the hair of a patient who had been taking arsenic medicinally (one-ninth of a grain per diem) it was found in the proportion of 0 3 in 10,000, and the same proportion was found in a patient suffering from well marked arsenical beer poisoning, while in another beer poisoning case arsenic was found in the hair in the proportion of 1 in 10,000. In control experiments made on the hair of healthy individuals, although arsenic was shown to be present in traces, it existed in quantities too minute to be estimated in one gramme of substance. Clearly this gives a hint which may be of service in elucidating the nature of obscure cases. All the same, its indications must be accepted with certain limitations, especially in medico-legal cases. Apparently arsenic once deposited in the hair would remain there; and the more we know of the many hitherto unsuspected modes in which arsenic may obtain access to articles of food, the less becomes the value as evidence of crime of any such proof as examination of the hair will offer of bygone arsenical infiltration.

Commenting on the above article, Dr. A. Dupré[2] says that some years ago in experimenting on the diffusion of various substances into and out of the animal tissues, he made use of chloride of lithium, and found that when this salt was administered the metal lithium was present in the hair, as well as in other tissues of the body. With the object of finding out whether this was the result of (1) direct diffusion into the hair; (2) absorption from the perspiration of the skin of the scalp; or (3) absorption from the hand passed through the hair, he carefully isolated some locks of hair by wrapping them in waterproof material, so as to prevent absorption by either of the last two possible means. Lithium, however, was never found in the hair so protected, and he came to the conclusion therefore that lithium did not diffuse directly into the hair. The question then is what would be the result in regard to arsenic were the same precautions taken.

[1] Lancet, March 23. [2] Lancet, April 6.

